# Crystal structure and Hirshfeld surface analysis of *N*-(2-chloro­phenyl­carbamo­thio­yl)-4-fluoro­benzamide and *N*-(4-bromo­phenyl­carbamo­thio­yl)-4-fluoro­benzamide

**DOI:** 10.1107/S2056989019008569

**Published:** 2019-06-21

**Authors:** Sidra Akhter, Muhammad Iqbal Choudhary, Hina Siddiqui, Sammer Yousuf

**Affiliations:** a H.E.J. Research Institute Of Chemistry, International Center for Chemical and Biological Sciences, University of Karachi, Karachi 75270, Pakistan

**Keywords:** Thio­urea, benzamide derivative, crystal structure, Hirshfeld surface

## Abstract

The title compounds, C_14_H_10_ClFN_2_OS (**1**) and C_14_H_10_BrFN_2_OS (**2**), were synthesized by two-step reactions. The dihedral angles between the aromatic rings are 31.99 (3) and 9.17 (5)° for **1** and **2**, respectively. Compound **1** features an intra­molecular bifurcated N—H⋯(O,Cl) link due to the presence of the *ortho*-Cl atom on the benzene ring, whereas **2** features an intra­molecular N—H⋯O hydrogen bond. In the crystal of **1**, inversion dimers linked by pairs of N—H⋯S hydrogen bonds generate *R*_2_^2^(8) loops. The extended structure of **2** features the same motif but an additional weak C—H⋯S inter­action links the inversion dimers into [100] double columns. Hirshfeld surface analyses indicate that the most important contributors towards the crystal packing are H⋯H (26.6%), S⋯H/H.·S (13.8%) and Cl⋯H/H⋯Cl (9.5%) contacts for **1** and H⋯H (19.7%), C⋯H/H⋯C (14.8%) and Br⋯H/H⋯Br (12.4%) contacts for **2**.

## Abstract 2

The title compounds, C_14_H_10_ClFN_2_OS (**1**) and C_14_H_10_BrFN_2_OS (**2**), were synthesized by two-step reactions·In the crystal of **1**, inversion dimers linked by pairs of N—H⋯S hydrogen bonds generate 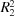
(8) loops. The extended structure of **2** features the same motif but an additional weak C—H⋯S inter­action links the inversion dimers into [100] double columns

## Chemical context   

Thio­urea and its derivatives show a broad range of biological activities (Solmaz *et al.*, 2018 ▸; Saeed *et al.*, 2018 ▸; Pandey *et al.*, 2019 ▸). The crystal structures of many thio­urea derivatives and their metal complexes have been reported (Lai *et al.*,2018 ▸; Contreras Aguilar *et al.*, 2018 ▸; Fakhar *et al.*, 2018 ▸; Mitoraj *et al.*, 2018 ▸; Pervez *et al.*, 2018 ▸; Hashim *et al.*, 2017 ▸ Ghazal *et al.*, 2019 ▸; Zhang *et al.*, 2019 ▸). As part of our studies in this area, we now describe the syntheses, crystal structures and Hirshfeld surface analyses of the thio­urea derivatives *N*-(2-chloro­phenyl­carbamo­thio­yl)-4-fluoro­benzamide (C_14_H_10_ClFN_2_OS, **1**) and *N*-(4-bromo­phenyl­carbamo­thio­yl)-4-fluoro­benzamide (C_14_H_10_BrFN_2_OS, **2**). The biological activities of these compounds were previously reported by Khan *et al.* (2018 ▸).
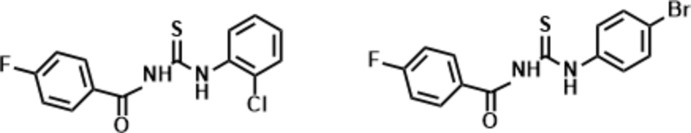


## Structural commentary   

**Figure 1:**
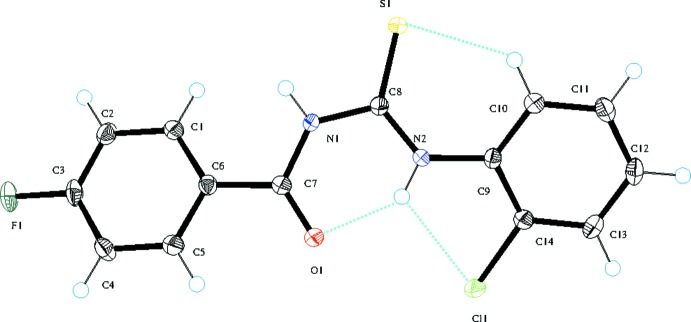
The mol­ecular structure of **1** showing 50% displacement ellipsoids; the blue lines represent the intra­molecular inter­actions.

**Figure 2:**
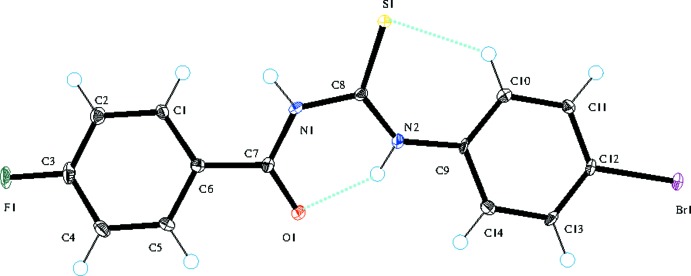
The mol­ecular structure of **2** showing 50% displacement ellipsoids; the blue lines represent the intra­molecular inter­actions.

Compound **1** (Fig. 1 ▸) is composed of a *para*-fluoro-substituted [C—F = 1.3579 (16) Å] benzoyl ring linked to a *ortho*-chloro-substituted phenyl ring [C—Cl = 1.7387 (14) Å] in while in **2** (Fig. 2 ▸), a *para*-fluoro-substituted [C—F = 1.350 (2) Å] benzoyl ring is linked to a *para*-bromo-substituted phenyl ring [C—Br = 1.8991 (17) Å] *via* a thio­urea (S1/N1/N2/C8) linkage. The benzoyl (O1/C1–C7) and phenyl rings (C9–C14) are arranged about the thio­urea moiety in an *anti* fashion having torsion angles C8—N1—C7—C6 = −170.22 (13) and C9—N2—C8—S1 = 4.5 (2)° in compound **1**, with corresponding values of −176.01 (16) and 3.8 (3)°, respectively, in compound **2**. The dihedral angles between the phenyl rings are 31.99 (3) and 9.17 (5)° in **1** and **2**, respectively. Compound **1** features an intra­moleclar bifurcated N—H⋯(O,Cl) hydrogen bond (Table 1 ▸) due to the presence of the *ortho*-Cl atom whereas **2** has an intra­molecular N—H⋯O link (Table 2 ▸). Both structures feature an intra­molecular C—H⋯S bond, which closes an *S*(6) ring. These intra­molecular hydrogen bonds may be responsible for the *anti* arrangement of the aromatic rings about the thio­urea linker.

## Supra­molecular features   

**Figure 3:**
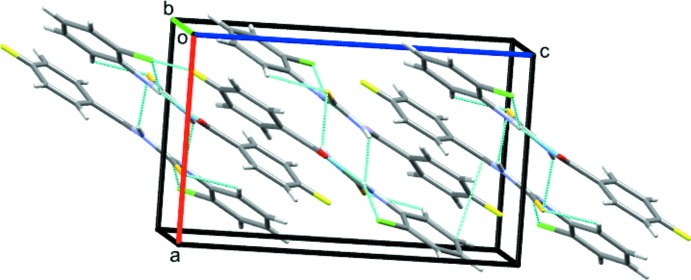
Partial packing diagram for **1**. Light-blue lines indicate directional inter­actions

**Figure 4:**
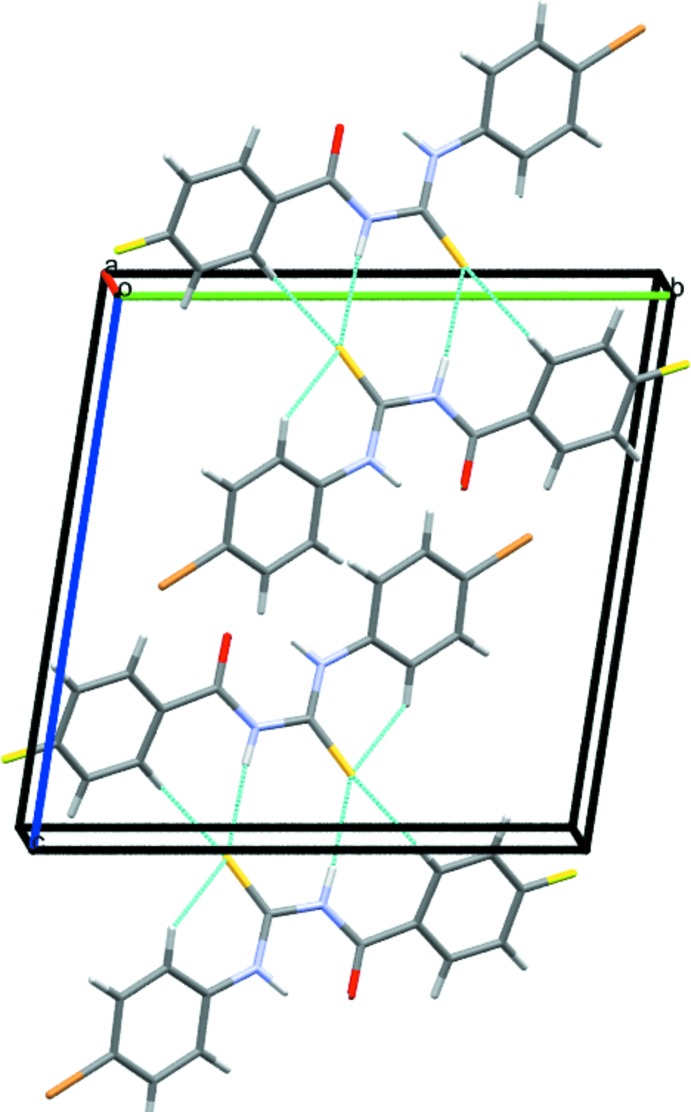
Partial packing diagram for **2**. Light-blue lines indicate directional inter­actions

In the crystal of **1**, inversion dimers linked by pairwise N1—H1*A*⋯S1 hydrogen bonds (Table 1 ▸) generate 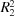
(8) loops (Fig. 3 ▸). The crystal of **2** features the same motif (Table 2 ▸), but an additional weak C—H⋯S bond links the dimers into double columns propagating in the [100] direction (Fig. 4 ▸).

## Database survey   

A search of Cambridge Structural Database (CSD version 5.39, update of February 2018) for compounds related to **1** and **2** yielded hits for *N*-{[4-chloro-3-(tri­fluoro­meth­yl)phen­yl]carbamo­thio­yl}-3-methyl­benzamide (CCDC deposition No. 1840069) and 4-chloro-*N*-{[4-chloro-3-(tri­fluoro­meth­yl)phen­yl]carbamo­thio­yl}benzamide (CCDC 1587395) (Zhang *et al.*, 2019 ▸): these compounds have the same skeleton as the title compounds but with different substituents attached to the phenyl rings. In both compounds, pairwise N—H⋯S hydrogen bonds are responsible for the formation of inversion dimers with an 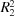
(8) motif, as also observed in title compounds.

## Hirshfeld surface analysis   

**Figure 5:**
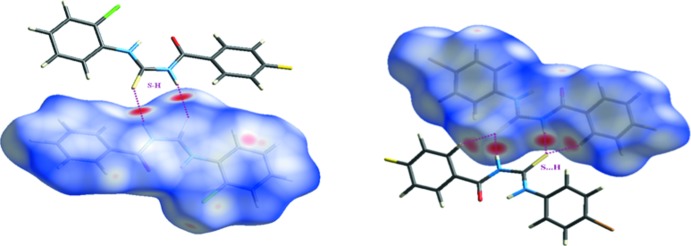
The Hirshfeld surfaces of **1** and **2**.

**Figure 6:**
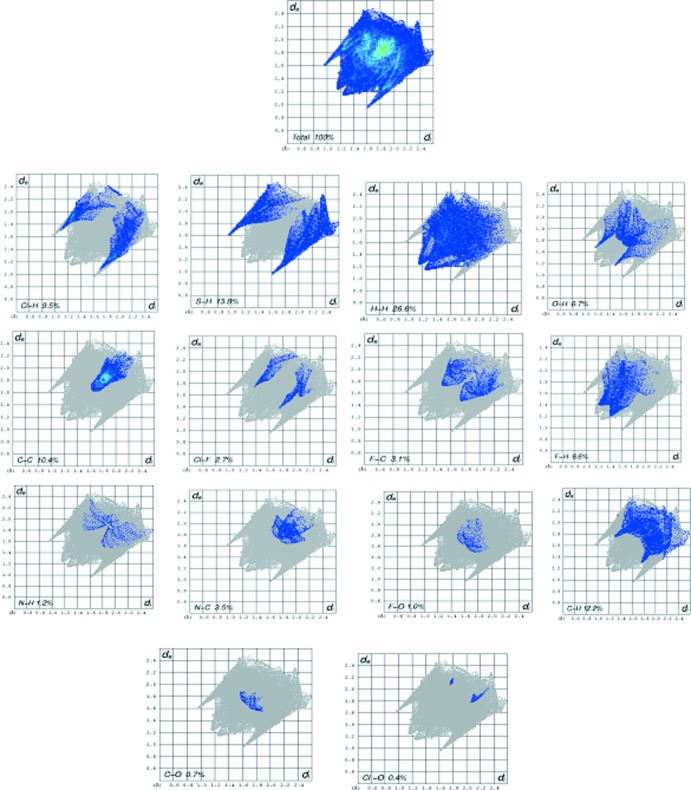
Two dimensional fingerprint plots for **1**.

In order to further analyse the close contacts and inter­molecular inter­actions in the crystals of **1** and **2**, Hirshfeld surfaces (mapped over *d*_norm_, curvedness and shape-index) (Fig. 5 ▸) and two-dimensional fingerprint plots (Figs. 6 ▸ and 7 ▸) were generated using *CrystalExplorer3.1* (Mackenzie *et al.*, 2017 ▸). The fingerprint plot for **1** decomposed into individual contact types indicates that the the most significant contributions are from H⋯H (van der Waals) (26.6%) contacts, followed by S⋯H/H⋯S (13.8%), Cl⋯H/H⋯Cl (9.5%) O⋯H/H⋯O (6.7%), F⋯H/H⋯F (6.6%), Cl⋯F/F⋯Cl (3.7%) and F⋯C/C⋯F (3.1%) inter­actions. In compound **2**, H⋯H (19.7%) (van der Waals contacts) are the most significant, followed by C⋯H/H⋯C (14.8%), S⋯H/H⋯S (12.6%), Br⋯H/H⋯Br (12.4%), C⋯C (9.9%) and O⋯N/N⋯O (7.9%) inter­actions.

## Synthesis and Crystallization   

Compounds **1** and **2** were synthesized by adopting a literature procedure (Binzet *et al.*, 2018 ▸) with slight modification: we refluxed the reactants in distilled solvents for 20 min. instead of refluxing them in anhydrous solvents for 4 h. In the first step, 4-fluoro­benzoyle chloride (1 mmol) and potassium thio­cyanate (1 mmol) were dissolved in acetone (10 ml) at room temperature with constant stirring for 20 minutes to obtain a white precipitate of 4-fluoro­phenyl iso­thio­cyanate. In the second step, 1 mmol of 2-chloro phenyl aniline (for **1**) or 4-bromo­phenyl aniline (for **2**) were added to the mixture and refluxed at 343 K. Hydro­chloric acid (0.5 *N*, 10 ml) was added and the solution was filtered to obtain the desired products: **1** in 69% yield and **2** in 80% yield. For recrystallization, compound **1** was dissolved in a mixture of di­chloro­methane and methanol (1:1) while compound **2** was dissolved in di­chloro­methane and left for slow evaporation at room temperature to obtain colourless prisms of **1** and colourless plates of **2**

## Data collection and Refinement   

Crystal data, data collection and structure refinement details are summarized in Table 3 ▸. The C-bound H atoms atoms were positioned with idealized geometry (C—H = 0.93–0.97 Å) and refined as riding atoms. In **1**, the N-bound H atoms were located in difference-Fourier maps and their positions were freely refined; in **2**, the N-bound H atoms were located in difference-Fourier maps and refined as riding atoms in their as-found relative positions. The constraint *U*_iso_(H) = 1.2*U*_eq_(carrier) was applied in all cases.

## Supplementary Material

Crystal structure: contains datablock(s) global, 1, 2. DOI: 10.1107/S2056989019008569/hb7804sup1.cif

Supporting information file. DOI: 10.1107/S2056989019008569/hb78041sup4.cml

Structure factors: contains datablock(s) 1. DOI: 10.1107/S2056989019008569/hb78041sup4.hkl

Structure factors: contains datablock(s) 2. DOI: 10.1107/S2056989019008569/hb78042sup3.hkl

Supporting information file. DOI: 10.1107/S2056989019008569/hb78042sup5.cml

CCDC references: 1923234, 1923233

Additional supporting information:  crystallographic information; 3D view; checkCIF report

## Figures and Tables

**Figure 7:**
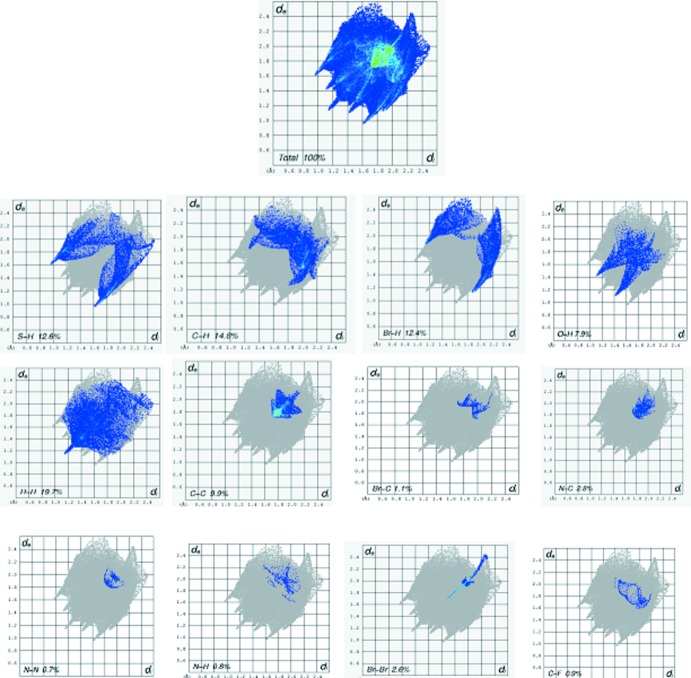
Two dimensional fingerprint plots for **2**.

**Table 1:** Hydrogen-bond geometry (Å, °) for **1** [Chem scheme1]

*D*—H⋯*A*	*D*—H	H⋯*A*	*D*⋯*A*	*D*—H⋯*A*
C10—H10⋯S1	0.95	2.57	3.1945 (14)	124
N2—H1*B*⋯Cl	0.87 (2)	2.482 (19)	2.9246 (12)	112.3 (14)
N2—H1*B*⋯O1	0.87 (2)	1.924 (19)	2.6600 (14)	141.6 (17)
N1—H1*A*⋯S1^i^	0.85 (2)	2.67 (2)	3.4031 (13)	145.2 (16)

Symmetry code: (i) 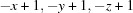.

**Table 2:** Hydrogen-bond geometry (Å, °) for **2** [Chem scheme1]

*D*—H⋯*A*	*D*—H	H⋯*A*	*D*⋯*A*	*D*—H⋯*A*
N1—H1*A*⋯S1^i^	0.88	2.69	3.5081 (15)	154
N2—H1*B*⋯O1	0.88	1.88	2.610 (2)	139
C10—H10⋯S1	0.95	2.65	3.2319 (18)	120
C1—H1⋯S1^ii^	0.95	2.81	3.7312 (18)	165

Symmetry codes: (i) 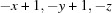; (ii) 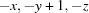.

**Table 3:** Experimental details

	**1**	**2**
Crystal data		
Chemical formula	C_14_H_10_ClFN_2_OS	C_14_H_10_BrFN_2_OS
*M*_r_	308.75	353.19
Crystal system, space group	Monoclinic, *P*2_1_/*c*	Triclinic, *P* 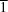
Temperature (K)	100	100
*a*, *b*, *c* (Å)	8.0785 (2), 12.4230 (3), 13.0772 (3)	3.8733 (2), 13.0776 (5), 13.2628 (6)
α, β, γ (°)	90, 90.551 (1), 90	98.817 (1), 94.714 (1), 94.727 (1)
*V* (Å^3^)	1312.36 (5)	658.54 (5)
*Z*	4	2
Radiation type	Cu *K*α	Cu *K*α
μ (mm^−1^)	4.15	5.83
Crystal size (mm)	0.11 × 0.07 × 0.03	0.35 × 0.05 × 0.04

Data collection		
Diffractometer	Bruker APEXII CCD	Bruker APEXII CCD
Absorption correction	Multi-scan (*SADABS*; Bruker, 2000 ▸)	Multi-scan (*SADABS*; Bruker, 2000 ▸)
*T*_min_, *T*_max_	0.682, 0.895	0.612, 0.946
No. of measured, independent and observed [*I* > 2σ(*I*)] reflections	18686, 2362, 2269	20701, 2384, 2381
*R*_int_	0.023	0.025
(sin θ/λ)_max_ (Å^−1^)	0.602	0.602

Refinement		
*R*[*F* ^2^ > 2σ(*F* ^2^)], *wR*(*F* ^2^), *S*	0.025, 0.069, 1.05	0.025, 0.110, 1.10
No. of reflections	2362	2384
No. of parameters	189	181
H-atom treatment	H atoms treated by a mixture of independent and constrained refinement	H-atom parameters constrained
Δρ_max_, Δρ_min_ (e Å^−3^)	0.22, −0.25	0.45, −1.46

Computer programs: *APEX2* and *SAINT* (Bruker, 2000 ▸), *SHELXT2014* (Sheldrick, 2015 ▸a), *SHELXL2016* (Sheldrick, 2015 ▸b) and *SHELXTL* (Sheldrick, 2008 ▸).

## supplementary crystallographic information

### N-(2-Chlorophenylcarbamothioyl)-4-fluorobenzamide (1) Crystal data

**Table:**

C_14_H_10_ClFN_2_OS	*F*(000) = 632
*M**_r_* = 308.75	*D*_x_ = 1.563 Mg m^−^^3^
Monoclinic, *P*2_1_/*c*	Cu *K*α radiation, λ = 1.54178 Å
*a* = 8.0785 (2) Å	Cell parameters from 9977 reflections
*b* = 12.4230 (3) Å	θ = 4.9–68.3°
*c* = 13.0772 (3) Å	µ = 4.15 mm^−^^1^
β = 90.551 (1)°	*T* = 100 K
*V* = 1312.36 (5) Å^3^	Prism, colourless
*Z* = 4	0.11 × 0.07 × 0.03 mm

### N-(2-Chlorophenylcarbamothioyl)-4-fluorobenzamide (1) Data collection

**Table:**

Bruker APEXII CCD diffractometer	2269 reflections with *I* > 2σ(*I*)
ω scans	*R*_int_ = 0.023
Absorption correction: multi-scan (SADABS; Bruker, 2000)	θ_max_ = 68.3°, θ_min_ = 4.9°
*T*_min_ = 0.682, *T*_max_ = 0.895	*h* = −9→9
18686 measured reflections	*k* = −14→14
2362 independent reflections	*l* = −15→15

### N-(2-Chlorophenylcarbamothioyl)-4-fluorobenzamide (1) Refinement

**Table:**

Refinement on *F*^2^	Primary atom site location: dual
Least-squares matrix: full	Hydrogen site location: mixed
*R*[*F*^2^ > 2σ(*F*^2^)] = 0.025	H atoms treated by a mixture of independent and constrained refinement
*wR*(*F*^2^) = 0.069	*w* = 1/[σ^2^(*F*_o_^2^) + (0.0347*P*)^2^ + 0.7594*P*] where *P* = (*F*_o_^2^ + 2*F*_c_^2^)/3
*S* = 1.05	(Δ/σ)_max_ = 0.001
2362 reflections	Δρ_max_ = 0.22 e Å^−^^3^
189 parameters	Δρ_min_ = −0.24 e Å^−^^3^
0 restraints	

### N-(2-Chlorophenylcarbamothioyl)-4-fluorobenzamide (1) Special details

**Table:**

Geometry. All esds (except the esd in the dihedral angle between two l.s. planes) are estimated using the full covariance matrix. The cell esds are taken into account individually in the estimation of esds in distances, angles and torsion angles; correlations between esds in cell parameters are only used when they are defined by crystal symmetry. An approximate (isotropic) treatment of cell esds is used for estimating esds involving l.s. planes.

### N-(2-Chlorophenylcarbamothioyl)-4-fluorobenzamide (1) Fractional atomic coordinates and isotropic or equivalent isotropic displacement parameters (Å^2^)

**Table:**

	*x*	*y*	*z*	*U*_iso_*/*U*_eq_	
S1	0.73325 (5)	0.47025 (3)	0.56374 (3)	0.02331 (12)	
Cl	0.83162 (5)	0.03157 (3)	0.58416 (3)	0.02495 (11)	
F1	0.18907 (12)	0.30493 (8)	0.01782 (7)	0.0314 (2)	
O1	0.54889 (12)	0.15637 (8)	0.42244 (7)	0.0192 (2)	
N1	0.56777 (15)	0.33824 (10)	0.44484 (9)	0.0179 (3)	
N2	0.70992 (14)	0.25311 (9)	0.57516 (9)	0.0157 (2)	
C1	0.43467 (17)	0.36712 (11)	0.24179 (10)	0.0177 (3)	
H1	0.493438	0.425890	0.271153	0.021*	
C2	0.35330 (18)	0.38022 (12)	0.14870 (11)	0.0206 (3)	
H2	0.355095	0.447218	0.113699	0.025*	
C3	0.26979 (18)	0.29291 (12)	0.10866 (10)	0.0211 (3)	
C4	0.26446 (18)	0.19359 (12)	0.15557 (11)	0.0224 (3)	
H4	0.206152	0.135158	0.125243	0.027*	
C5	0.34669 (18)	0.18165 (11)	0.24822 (11)	0.0199 (3)	
H5	0.345811	0.113881	0.281860	0.024*	
C6	0.43099 (16)	0.26820 (11)	0.29281 (10)	0.0156 (3)	
C7	0.51917 (16)	0.24761 (11)	0.39152 (10)	0.0160 (3)	
C8	0.67071 (16)	0.34671 (11)	0.53083 (10)	0.0162 (3)	
C9	0.80473 (16)	0.23264 (11)	0.66453 (10)	0.0148 (3)	
C10	0.83157 (17)	0.30697 (11)	0.74300 (10)	0.0184 (3)	
H10	0.789298	0.378033	0.736600	0.022*	
C11	0.91962 (17)	0.27772 (12)	0.83031 (11)	0.0213 (3)	
H11	0.938625	0.329427	0.882642	0.026*	
C12	0.98017 (18)	0.17407 (12)	0.84215 (11)	0.0224 (3)	
H12	1.040289	0.154849	0.902214	0.027*	
C13	0.95246 (18)	0.09856 (12)	0.76576 (11)	0.0217 (3)	
H13	0.992398	0.027057	0.773432	0.026*	
C14	0.86603 (17)	0.12824 (11)	0.67813 (10)	0.0170 (3)	
H1B	0.672 (2)	0.1970 (16)	0.5432 (14)	0.028 (5)*	
H1A	0.528 (2)	0.3988 (16)	0.4260 (14)	0.031 (5)*	

### N-(2-Chlorophenylcarbamothioyl)-4-fluorobenzamide (1) Atomic displacement parameters (Å^2^)

**Table:**

	*U*^11^	*U*^22^	*U*^33^	*U*^12^	*U*^13^	*U*^23^
S1	0.0331 (2)	0.01172 (18)	0.0249 (2)	−0.00054 (13)	−0.01311 (15)	−0.00095 (12)
Cl	0.0406 (2)	0.01417 (18)	0.01996 (19)	0.00501 (14)	−0.00550 (15)	−0.00194 (12)
F1	0.0374 (5)	0.0388 (5)	0.0178 (4)	0.0005 (4)	−0.0147 (4)	0.0016 (4)
O1	0.0267 (5)	0.0138 (5)	0.0170 (5)	−0.0006 (4)	−0.0048 (4)	0.0011 (4)
N1	0.0248 (6)	0.0126 (6)	0.0161 (6)	0.0039 (5)	−0.0079 (5)	−0.0007 (4)
N2	0.0206 (6)	0.0125 (6)	0.0141 (6)	0.0000 (5)	−0.0046 (4)	−0.0015 (4)
C1	0.0208 (7)	0.0165 (6)	0.0158 (7)	0.0002 (5)	−0.0008 (5)	−0.0019 (5)
C2	0.0257 (7)	0.0196 (7)	0.0166 (7)	0.0034 (6)	−0.0016 (6)	0.0024 (5)
C3	0.0209 (7)	0.0304 (8)	0.0119 (7)	0.0041 (6)	−0.0042 (5)	−0.0015 (6)
C4	0.0241 (7)	0.0243 (8)	0.0188 (7)	−0.0040 (6)	−0.0045 (6)	−0.0048 (6)
C5	0.0240 (7)	0.0170 (7)	0.0187 (7)	−0.0012 (6)	−0.0017 (5)	−0.0007 (5)
C6	0.0158 (6)	0.0171 (7)	0.0137 (6)	0.0017 (5)	−0.0004 (5)	−0.0012 (5)
C7	0.0171 (6)	0.0152 (7)	0.0158 (7)	0.0000 (5)	−0.0002 (5)	−0.0011 (5)
C8	0.0184 (6)	0.0150 (6)	0.0152 (6)	0.0020 (5)	−0.0023 (5)	−0.0010 (5)
C9	0.0139 (6)	0.0169 (6)	0.0135 (6)	−0.0009 (5)	−0.0008 (5)	0.0022 (5)
C10	0.0208 (7)	0.0169 (7)	0.0175 (7)	−0.0002 (5)	−0.0020 (5)	−0.0003 (5)
C11	0.0221 (7)	0.0250 (7)	0.0167 (7)	−0.0030 (6)	−0.0034 (5)	−0.0019 (6)
C12	0.0211 (7)	0.0292 (8)	0.0169 (7)	0.0001 (6)	−0.0055 (5)	0.0048 (6)
C13	0.0226 (7)	0.0211 (7)	0.0215 (7)	0.0039 (6)	−0.0024 (6)	0.0053 (6)
C14	0.0189 (6)	0.0160 (7)	0.0161 (6)	0.0000 (5)	−0.0001 (5)	−0.0007 (5)

### N-(2-Chlorophenylcarbamothioyl)-4-fluorobenzamide (1) Geometric parameters (Å, º)

**Table:**

S1—C8	1.6709 (14)	C4—C5	1.384 (2)
Cl—C14	1.7387 (14)	C4—H4	0.9500
F1—C3	1.3579 (16)	C5—C6	1.3972 (19)
O1—C7	1.2264 (17)	C5—H5	0.9500
N1—C7	1.3794 (18)	C6—C7	1.4904 (18)
N1—C8	1.3961 (17)	C9—C10	1.3959 (19)
N1—H1A	0.85 (2)	C9—C14	1.3990 (19)
N2—C8	1.3360 (18)	C10—C11	1.3879 (19)
N2—C9	1.4141 (17)	C10—H10	0.9500
N2—H1B	0.87 (2)	C11—C12	1.385 (2)
C1—C2	1.3875 (19)	C11—H11	0.9500
C1—C6	1.3987 (19)	C12—C13	1.387 (2)
C1—H1	0.9500	C12—H12	0.9500
C2—C3	1.378 (2)	C13—C14	1.3860 (19)
C2—H2	0.9500	C13—H13	0.9500
C3—C4	1.379 (2)		
			
C7—N1—C8	129.28 (12)	O1—C7—N1	122.26 (12)
C7—N1—H1A	117.8 (13)	O1—C7—C6	122.32 (12)
C8—N1—H1A	112.8 (13)	N1—C7—C6	115.41 (12)
C8—N2—C9	129.75 (12)	N2—C8—N1	114.89 (12)
C8—N2—H1B	114.1 (12)	N2—C8—S1	128.15 (10)
C9—N2—H1B	116.1 (12)	N1—C8—S1	116.94 (10)
C2—C1—C6	120.64 (13)	C10—C9—C14	117.87 (12)
C2—C1—H1	119.7	C10—C9—N2	124.58 (12)
C6—C1—H1	119.7	C14—C9—N2	117.39 (12)
C3—C2—C1	117.84 (13)	C11—C10—C9	120.42 (13)
C3—C2—H2	121.1	C11—C10—H10	119.8
C1—C2—H2	121.1	C9—C10—H10	119.8
F1—C3—C2	118.37 (13)	C12—C11—C10	120.86 (13)
F1—C3—C4	118.09 (13)	C12—C11—H11	119.6
C2—C3—C4	123.54 (13)	C10—C11—H11	119.6
C3—C4—C5	117.94 (13)	C11—C12—C13	119.58 (13)
C3—C4—H4	121.0	C11—C12—H12	120.2
C5—C4—H4	121.0	C13—C12—H12	120.2
C4—C5—C6	120.72 (13)	C14—C13—C12	119.47 (13)
C4—C5—H5	119.6	C14—C13—H13	120.3
C6—C5—H5	119.6	C12—C13—H13	120.3
C5—C6—C1	119.30 (12)	C13—C14—C9	121.78 (13)
C5—C6—C7	117.15 (12)	C13—C14—Cl	118.45 (11)
C1—C6—C7	123.49 (12)	C9—C14—Cl	119.76 (10)
			
C6—C1—C2—C3	−0.1 (2)	C9—N2—C8—S1	4.5 (2)
C1—C2—C3—F1	179.70 (12)	C7—N1—C8—N2	−9.6 (2)
C1—C2—C3—C4	−0.7 (2)	C7—N1—C8—S1	168.84 (11)
F1—C3—C4—C5	−179.92 (12)	C8—N2—C9—C10	22.4 (2)
C2—C3—C4—C5	0.5 (2)	C8—N2—C9—C14	−162.20 (13)
C3—C4—C5—C6	0.6 (2)	C14—C9—C10—C11	1.4 (2)
C4—C5—C6—C1	−1.3 (2)	N2—C9—C10—C11	176.73 (13)
C4—C5—C6—C7	−178.72 (13)	C9—C10—C11—C12	−1.1 (2)
C2—C1—C6—C5	1.1 (2)	C10—C11—C12—C13	0.0 (2)
C2—C1—C6—C7	178.32 (13)	C11—C12—C13—C14	0.7 (2)
C8—N1—C7—O1	8.6 (2)	C12—C13—C14—C9	−0.3 (2)
C8—N1—C7—C6	−170.22 (13)	C12—C13—C14—Cl	−179.45 (11)
C5—C6—C7—O1	15.54 (19)	C10—C9—C14—C13	−0.7 (2)
C1—C6—C7—O1	−161.73 (13)	N2—C9—C14—C13	−176.39 (12)
C5—C6—C7—N1	−165.60 (12)	C10—C9—C14—Cl	178.42 (10)
C1—C6—C7—N1	17.14 (19)	N2—C9—C14—Cl	2.72 (17)
C9—N2—C8—N1	−177.23 (12)		

### N-(2-Chlorophenylcarbamothioyl)-4-fluorobenzamide (1) Hydrogen-bond geometry (Å, º)

**Table:**

*D*—H···*A*	*D*—H	H···*A*	*D*···*A*	*D*—H···*A*
C10—H10···S1	0.95	2.57	3.1945 (14)	124
N2—H1*B*···Cl	0.87 (2)	2.482 (19)	2.9246 (12)	112.3 (14)
N2—H1*B*···O1	0.87 (2)	1.924 (19)	2.6600 (14)	141.6 (17)
N1—H1*A*···S1^i^	0.85 (2)	2.67 (2)	3.4031 (13)	145.2 (16)

Symmetry code: (i) −*x*+1, −*y*+1, −*z*+1.

### N-(4-Bromophenylcarbamothioyl)-4-fluorobenzamide (2) Crystal data

**Table:**

C_14_H_10_BrFN_2_OS	*Z* = 2
*M**_r_* = 353.19	*F*(000) = 348
Triclinic, *P*1	*D*_x_ = 1.771 Mg m^−^^3^
*a* = 3.8733 (2) Å	Cu *K*α radiation, λ = 1.54178 Å
*b* = 13.0776 (5) Å	Cell parameters from 9945 reflections
*c* = 13.2628 (6) Å	θ = 3.4–68.2°
α = 98.817 (1)°	µ = 5.83 mm^−^^1^
β = 94.714 (1)°	*T* = 100 K
γ = 94.727 (1)°	Plate, colourless
*V* = 658.54 (5) Å^3^	0.35 × 0.05 × 0.04 mm

### N-(4-Bromophenylcarbamothioyl)-4-fluorobenzamide (2) Data collection

**Table:**

Bruker APEXII CCD diffractometer	2381 reflections with *I* > 2σ(*I*)
φ and ω scans	*R*_int_ = 0.025
Absorption correction: multi-scan (SADABS; Bruker, 2000)	θ_max_ = 68.2°, θ_min_ = 3.4°
*T*_min_ = 0.612, *T*_max_ = 0.946	*h* = −4→4
20701 measured reflections	*k* = −15→15
2384 independent reflections	*l* = −15→15

### N-(4-Bromophenylcarbamothioyl)-4-fluorobenzamide (2) Refinement

**Table:**

Refinement on *F*^2^	Primary atom site location: dual
Least-squares matrix: full	Hydrogen site location: inferred from neighbouring sites
*R*[*F*^2^ > 2σ(*F*^2^)] = 0.025	H-atom parameters constrained
*wR*(*F*^2^) = 0.110	*w* = 1/[σ^2^(*F*_o_^2^) + (0.1*P*)^2^] where *P* = (*F*_o_^2^ + 2*F*_c_^2^)/3
*S* = 1.10	(Δ/σ)_max_ = 0.001
2384 reflections	Δρ_max_ = 0.45 e Å^−^^3^
181 parameters	Δρ_min_ = −1.46 e Å^−^^3^
0 restraints	

### N-(4-Bromophenylcarbamothioyl)-4-fluorobenzamide (2) Special details

**Table:**

Geometry. All esds (except the esd in the dihedral angle between two l.s. planes) are estimated using the full covariance matrix. The cell esds are taken into account individually in the estimation of esds in distances, angles and torsion angles; correlations between esds in cell parameters are only used when they are defined by crystal symmetry. An approximate (isotropic) treatment of cell esds is used for estimating esds involving l.s. planes.

### N-(4-Bromophenylcarbamothioyl)-4-fluorobenzamide (2) Fractional atomic coordinates and isotropic or equivalent isotropic displacement parameters (Å^2^)

**Table:**

	*x*	*y*	*z*	*U*_iso_*/*U*_eq_	
Br1	−0.39350 (4)	0.11758 (2)	0.46689 (2)	0.01272 (16)	
O1	0.4560 (4)	0.66092 (11)	0.32207 (10)	0.0175 (3)	
N1	0.3835 (4)	0.58122 (12)	0.15534 (11)	0.0106 (3)	
H1A	0.419261	0.590591	0.092584	0.013*	
N2	0.1544 (4)	0.47331 (12)	0.26027 (11)	0.0111 (3)	
H1B	0.187274	0.531645	0.304308	0.013*	
F1	0.9679 (3)	1.04344 (9)	0.12214 (9)	0.0205 (3)	
S1	0.18617 (10)	0.38973 (3)	0.06266 (3)	0.00989 (18)	
C1	0.5195 (4)	0.78724 (14)	0.09959 (14)	0.0092 (4)	
H1	0.376290	0.737919	0.050359	0.011*	
C2	0.6398 (5)	0.88103 (14)	0.07394 (14)	0.0129 (4)	
H2	0.581012	0.896819	0.007460	0.016*	
C3	0.8466 (5)	0.95143 (15)	0.14634 (15)	0.0146 (4)	
C4	0.9345 (5)	0.93323 (14)	0.24544 (14)	0.0137 (4)	
H4	1.074144	0.983856	0.294279	0.016*	
C5	0.8117 (5)	0.83897 (14)	0.27036 (14)	0.0116 (4)	
H5	0.866416	0.824564	0.337592	0.014*	
C6	0.6063 (5)	0.76392 (14)	0.19744 (13)	0.0101 (4)	
C7	0.4782 (5)	0.66558 (14)	0.23084 (14)	0.0105 (4)	
C9	0.0233 (4)	0.38653 (13)	0.30136 (13)	0.0086 (4)	
C8	0.2378 (4)	0.48253 (14)	0.16562 (13)	0.0085 (4)	
C10	−0.1837 (5)	0.30056 (14)	0.24536 (13)	0.0115 (4)	
H10	−0.240925	0.296827	0.173769	0.014*	
C11	−0.3051 (4)	0.22064 (14)	0.29513 (13)	0.0105 (4)	
H11	−0.444455	0.161739	0.257544	0.013*	
C12	−0.2224 (5)	0.22707 (13)	0.39998 (14)	0.0102 (4)	
C13	−0.0217 (5)	0.31221 (13)	0.45694 (13)	0.0109 (4)	
H13	0.031180	0.315998	0.528723	0.013*	
C14	0.0999 (5)	0.39136 (15)	0.40755 (14)	0.0114 (4)	
H14	0.237633	0.450144	0.445916	0.014*	

### N-(4-Bromophenylcarbamothioyl)-4-fluorobenzamide (2) Atomic displacement parameters (Å^2^)

**Table:**

	*U*^11^	*U*^22^	*U*^33^	*U*^12^	*U*^13^	*U*^23^
Br1	0.0169 (2)	0.0096 (2)	0.0125 (2)	−0.00227 (13)	0.00215 (13)	0.00588 (13)
O1	0.0312 (8)	0.0122 (7)	0.0076 (7)	−0.0065 (6)	0.0033 (6)	0.0011 (5)
N1	0.0154 (8)	0.0108 (8)	0.0057 (7)	0.0005 (6)	0.0007 (6)	0.0022 (6)
N2	0.0170 (8)	0.0073 (7)	0.0090 (7)	−0.0001 (6)	0.0021 (6)	0.0019 (6)
F1	0.0328 (7)	0.0078 (6)	0.0213 (7)	−0.0054 (5)	0.0078 (5)	0.0051 (5)
S1	0.0140 (3)	0.0082 (3)	0.0069 (3)	−0.00094 (19)	0.00084 (19)	0.00050 (19)
C1	0.0092 (8)	0.0080 (9)	0.0102 (8)	−0.0004 (6)	0.0023 (6)	0.0005 (7)
C2	0.0161 (9)	0.0103 (9)	0.0133 (9)	0.0022 (7)	0.0031 (7)	0.0031 (7)
C3	0.0197 (9)	0.0068 (9)	0.0194 (10)	0.0022 (7)	0.0119 (8)	0.0033 (7)
C4	0.0149 (9)	0.0097 (9)	0.0148 (9)	−0.0026 (7)	0.0020 (7)	−0.0021 (7)
C5	0.0144 (8)	0.0095 (9)	0.0093 (8)	−0.0016 (7)	−0.0006 (7)	−0.0012 (7)
C6	0.0106 (8)	0.0103 (9)	0.0089 (9)	−0.0002 (7)	0.0008 (6)	0.0008 (7)
C7	0.0118 (8)	0.0104 (9)	0.0098 (9)	−0.0013 (7)	0.0013 (6)	0.0037 (7)
C9	0.0120 (8)	0.0070 (9)	0.0086 (8)	0.0005 (7)	0.0048 (6)	0.0047 (6)
C8	0.0103 (8)	0.0086 (8)	0.0071 (8)	0.0020 (7)	−0.0002 (6)	0.0026 (6)
C10	0.0137 (8)	0.0120 (9)	0.0088 (8)	0.0001 (7)	−0.0005 (7)	0.0028 (6)
C11	0.0116 (8)	0.0092 (9)	0.0097 (8)	−0.0028 (6)	0.0007 (6)	0.0004 (6)
C12	0.0128 (8)	0.0080 (9)	0.0112 (8)	0.0005 (6)	0.0023 (7)	0.0056 (6)
C13	0.0138 (8)	0.0132 (9)	0.0067 (8)	−0.0003 (7)	0.0018 (7)	0.0048 (6)
C14	0.0130 (8)	0.0092 (9)	0.0112 (9)	−0.0007 (6)	0.0011 (7)	0.0002 (6)

### N-(4-Bromophenylcarbamothioyl)-4-fluorobenzamide (2) Geometric parameters (Å, º)

**Table:**

Br1—C12	1.8991 (17)	C4—C5	1.382 (3)
O1—C7	1.230 (2)	C4—H4	0.9500
N1—C7	1.374 (2)	C5—C6	1.409 (2)
N1—C8	1.396 (2)	C5—H5	0.9500
N1—H1A	0.8800	C6—C7	1.484 (3)
N2—C8	1.342 (2)	C9—C10	1.399 (2)
N2—C9	1.408 (2)	C9—C14	1.406 (3)
N2—H1B	0.8800	C10—C11	1.390 (3)
F1—C3	1.350 (2)	C10—H10	0.9500
S1—C8	1.6687 (18)	C11—C12	1.389 (2)
C1—C2	1.377 (3)	C11—H11	0.9500
C1—C6	1.399 (3)	C12—C13	1.385 (3)
C1—H1	0.9500	C13—C14	1.379 (3)
C2—C3	1.375 (3)	C13—H13	0.9500
C2—H2	0.9500	C14—H14	0.9500
C3—C4	1.391 (3)		
			
C7—N1—C8	128.20 (16)	O1—C7—N1	122.08 (17)
C7—N1—H1A	115.9	O1—C7—C6	121.00 (17)
C8—N1—H1A	115.9	N1—C7—C6	116.92 (16)
C8—N2—C9	131.19 (16)	C10—C9—C14	119.30 (16)
C8—N2—H1B	114.4	C10—C9—N2	124.87 (15)
C9—N2—H1B	114.4	C14—C9—N2	115.78 (16)
C2—C1—C6	120.62 (17)	N2—C8—N1	114.66 (16)
C2—C1—H1	119.7	N2—C8—S1	126.92 (15)
C6—C1—H1	119.7	N1—C8—S1	118.43 (13)
C3—C2—C1	118.79 (17)	C11—C10—C9	119.57 (16)
C3—C2—H2	120.6	C11—C10—H10	120.2
C1—C2—H2	120.6	C9—C10—H10	120.2
F1—C3—C2	119.46 (18)	C12—C11—C10	119.85 (16)
F1—C3—C4	117.55 (18)	C12—C11—H11	120.1
C2—C3—C4	122.98 (18)	C10—C11—H11	120.1
C5—C4—C3	117.70 (18)	C13—C12—C11	121.42 (16)
C5—C4—H4	121.1	C13—C12—Br1	119.31 (13)
C3—C4—H4	121.1	C11—C12—Br1	119.27 (13)
C4—C5—C6	120.91 (18)	C14—C13—C12	118.76 (16)
C4—C5—H5	119.5	C14—C13—H13	120.6
C6—C5—H5	119.5	C12—C13—H13	120.6
C1—C6—C5	118.97 (17)	C13—C14—C9	121.10 (17)
C1—C6—C7	123.33 (17)	C13—C14—H14	119.5
C5—C6—C7	117.62 (16)	C9—C14—H14	119.5
			
C6—C1—C2—C3	−0.1 (3)	C8—N2—C9—C10	−28.7 (3)
C1—C2—C3—F1	179.66 (16)	C8—N2—C9—C14	154.04 (18)
C1—C2—C3—C4	−1.4 (3)	C9—N2—C8—N1	−175.93 (16)
F1—C3—C4—C5	−179.80 (16)	C9—N2—C8—S1	3.8 (3)
C2—C3—C4—C5	1.2 (3)	C7—N1—C8—N2	6.3 (3)
C3—C4—C5—C6	0.4 (3)	C7—N1—C8—S1	−173.53 (14)
C2—C1—C6—C5	1.6 (3)	C14—C9—C10—C11	−1.1 (3)
C2—C1—C6—C7	178.38 (16)	N2—C9—C10—C11	−178.32 (16)
C4—C5—C6—C1	−1.8 (3)	C9—C10—C11—C12	0.5 (3)
C4—C5—C6—C7	−178.72 (16)	C10—C11—C12—C13	0.4 (3)
C8—N1—C7—O1	3.4 (3)	C10—C11—C12—Br1	179.54 (13)
C8—N1—C7—C6	−176.01 (16)	C11—C12—C13—C14	−0.6 (3)
C1—C6—C7—O1	−154.97 (17)	Br1—C12—C13—C14	−179.76 (13)
C5—C6—C7—O1	21.9 (3)	C12—C13—C14—C9	0.0 (3)
C1—C6—C7—N1	24.4 (2)	C10—C9—C14—C13	0.9 (3)
C5—C6—C7—N1	−158.75 (16)	N2—C9—C14—C13	178.36 (14)

### N-(4-Bromophenylcarbamothioyl)-4-fluorobenzamide (2) Hydrogen-bond geometry (Å, º)

**Table:**

*D*—H···*A*	*D*—H	H···*A*	*D*···*A*	*D*—H···*A*
N1—H1*A*···S1^i^	0.88	2.69	3.5081 (15)	154
N2—H1*B*···O1	0.88	1.88	2.610 (2)	139
C10—H10···S1	0.95	2.65	3.2319 (18)	120
C1—H1···S1^ii^	0.95	2.81	3.7312 (18)	165

Symmetry codes: (i) −*x*+1, −*y*+1, −*z*; (ii) −*x*, −*y*+1, −*z*.
